# Impact of hyaluronic acid-modified hafnium metalorganic frameworks containing rhynchophylline on Alzheimer’s disease

**DOI:** 10.1515/biol-2022-1043

**Published:** 2025-03-18

**Authors:** Shiguo Lin, Yanshan Ye, Sujin Lin

**Affiliations:** Department of Psychiatry, Wenzhou Seventh People’s Hospital, Wenzhou, Zhejiang, 325000, P. R. China; Department of Rehabilitation, Wenzhou TCM Hospital of Zhejiang Chinese Medical University, Wenzhou, Zhejiang, 325000, P. R. China

**Keywords:** neurodegenerative disorder, Alzheimer’s disease, amyloid beta, Rhynchophylline, metal-organic frameworks, tau protein phosphorylation

## Abstract

Rhynchophylline (Rhy) is an attractive candidate, harboring ameliorative effects on Alzheimer’s disease (AD). Nevertheless, its application is impeded by its low water solubility and poor bioavailability. Here we synthesized and characterized the Rhy-loaded hyaluronic acid-modified hafnium metal-organic frameworks (HA@Rhy@Hf-MOF). The drug release profiles of free Rhy from HA@Rhy@Hf-MOF were evaluated, and the cellular toxicity was assessed through Cell Counting Kit-8 (CCK-8) assay. *In vivo* experiments included behavioral experiments of various murine capabilities, with neuronal damage appraised through Hematoxylin and Eosin staining and Nissl staining. Subsequently, the formation of AD-related amyloid beta (Aβ) plaques formation and Tau phosphorylation were measured. The HA@Rhy@Hf-MOF with spherical shape were presented as uniformly dispersed and with a negative charge, exhibiting a pronounced pharmacological sustained-release effect and minimal cellular toxicity. Findings from the Morris water maze test, novel object recognition test, and elevated plus maze test substantiated that HA@Rhy@Hf-MOF effectively mitigated cognitive deficiency and anxiety, and enhanced spatial learning in AD mice. Immunofluorescence staining and Western blot both illustrated that HA@Rhy@Hf-MOF could attenuate hippocampal Aβ formation and deposition, as well as tau hyperphosphorylation. In conclusion, HA@Rhy@Hf-MOF exerts its therapeutic efficacy against AD by targeting the deposition of Aβ plaques and inhibiting site-specific phosphorylation of Tau.

## 1 Introduction

Alzheimer’s disease (AD) constitutes a progressive neurodegenerative ailment predominantly afflicting individuals in the middle and advanced stages of life. Clinically, it frequently presents itself through cognitive impairment, memory regression, linguistic anomalies, emotional desuetude, and compromised motor proficiency, thereby rendering self-care a formidable challenge for the majority of AD patients, imperiling their very existence [1]. The primary neuropathological alterations of AD involve the development of neuritic plaques (the extracellular aggregation of amyloid beta [Aβ] peptides) and neurofibrillary tangles (the intracellular accumulation of hyperphosphorylated tau protein within neurons), coupled with progressive neuronal loss and cerebral atrophy. Currently available medications for AD primarily focus on symptom alleviation, but are not universally effective across all patients. Herbal extracts containing alkaloids, with a historical application spanning thousands of years in traditional medicine, and the utilization of naturally occurring alkaloids in the treatment of AD has garnered substantial interest [2].

*Uncaria rhynchophylla*, known as Gou-Teng in Chinese, has shown promise as an herbal remedy for AD. The extract derived from *Uncaria rhynchophylla* has exhibited potent anti-aggregation effects on Aβ proteins [3] and was validated in ameliorating cognitive impairments caused by d-galactose in mice [4]. Pharmacological investigations reveal that the alkaloids within the *Uncaria rhynchophylla* can exert neuroprotective effects and ameliorate cognitive impairments through a myriad of mechanisms. These alkaloids showcase the capacity to salvage compromised synaptic plasticity in the hippocampus and mitigate cognitive dysfunction in AD mice [5]. Furthermore, they could alleviate Aβ amyloid plaque burden and diminish inflammation in AD mice [6]. The alkaloid rhynchophylline (Rhy), extracted from the hooked branches of *Uncaria rhynchophylla*, possesses the ability to inhibit peripheral vascular constriction, causing reduced vascular resistance and blood pressure. Simultaneously, it exhibits antiplatelet aggregation and antithrombotic effects [7,8]. Moreover, Rhy possesses the capability to traverse the blood–brain barrier, fostering the development of intricate neuronal networks through the upregulation of neurogenesis [9]. These findings collectively underscore Rhy as a compelling candidate for eliciting ameliorative effects on AD. However, the application of Rhy in AD treatment is constrained by its low water solubility, low concentration in brain tissue, and poor bioavailability [10]. Therefore, there is an urgent need for a nanomaterial that can enhance blood–brain barrier permeability, and improve the water solubility and bioavailability of Rhy, thereby increasing the effectiveness of Rhy in treating AD.

Targeted therapeutic approaches for the multifaceted etiology of AD primarily encompass the inhibition of Aβ aggregation, promotion of Aβ clearance, reduction in oxidative stress, facilitation of neuronal regeneration, and so on. Additionally, imbalances in metal ions can lead to the deposition of Aβ proteins and subsequent neurotoxicity [11]. Some nanoparticles have been proven to target Aβ aggregation, but their intricate modification processes, lack of metal chelation capability, and poor protein enrichment abilities significantly restrict their further applications [12,13,14]. Metal-organic frameworks (MOF) are highly crystalline assemblies comprising metal ions or clusters and multidentate organic ligands. Owing to their excellent properties such as porousness, chemical versatility, and biodegradability, MOF hold expansive prospects in various domains such as catalysis, metal ion/gas storage, drug delivery, sensing, chemical separation, and biomedical imaging [15]. Among them, Hafnium-based MOF (Hf-MOF) has been reported as the optimal Aβ oxidant due to its superior ability to generate singlet oxygen (^1^O_2_). Its efficacy has been expounded upon in the AD nematode model, demonstrating a reduction in Aβ-induced cellular toxicity and an extension of the nematode lifespan [16]. Furthermore, hyaluronic acid (HA), a natural high-molecular-weight polysaccharide, serves as a vital component in the central nervous system and functions as a scaffold in the extracellular matrix. HA molecules exhibit intense hydrophilicity and can be rapidly degraded by hyaluronidase [17]. Consequently, the utilization of HA for the outer modification of Hf-MOF could significantly enhance the water solubility of the nanocarrier system and prolong its systemic circulation time, with the precise functionalities awaiting further confirmation.

**Figure 1:**
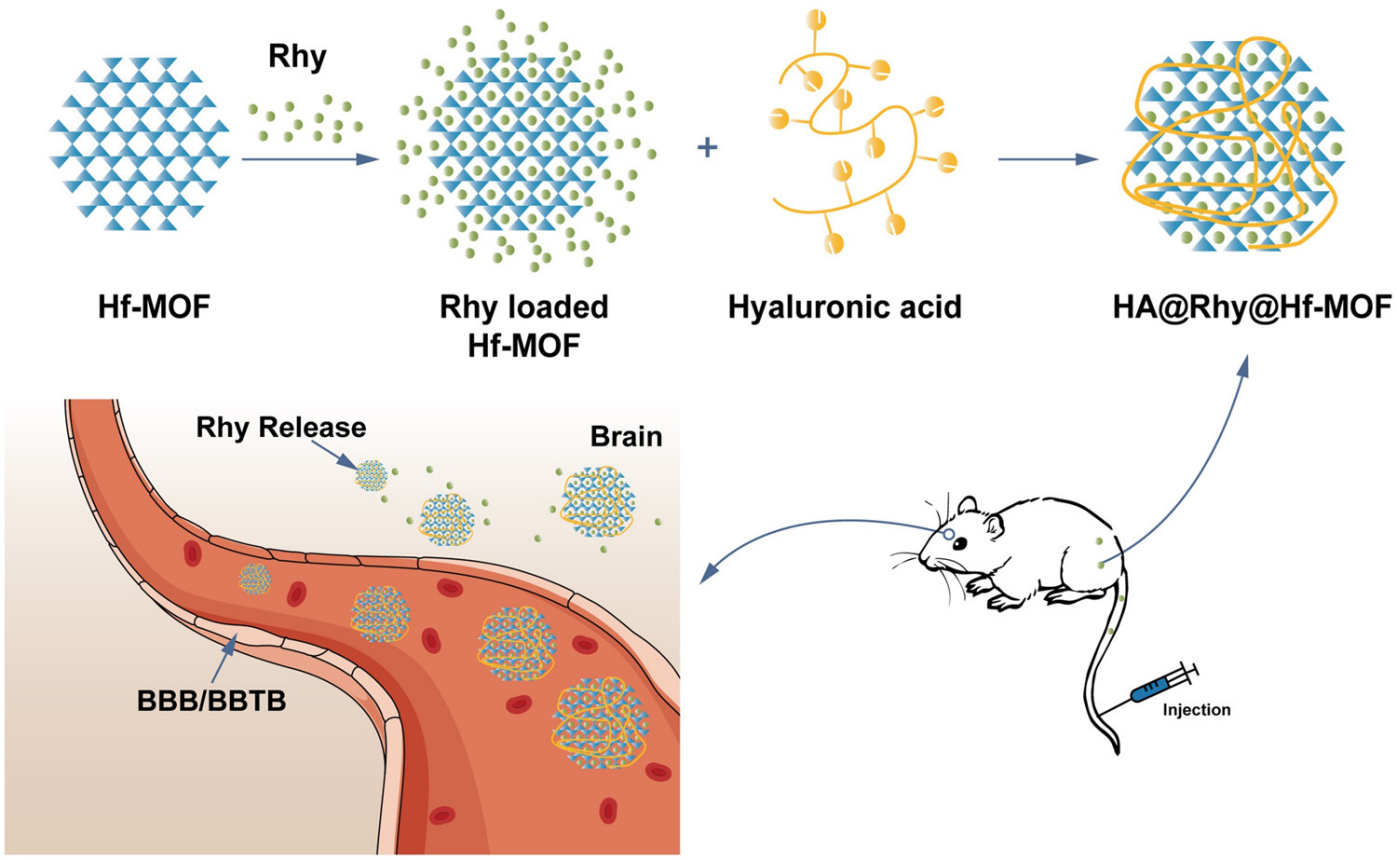
Synthesis and anti-AD activity research of HA@Rhy@Hf-MOF. BBB: blood–brain barrier.

In this investigation, we designed and established the Rhy-loaded HA-modified Hf-MOF (HA@Rhy@Hf-MOF), with Hf-MOF to boost the bioavailability and HA to improve the water solubility of Rhy. The characterization of this newly synthesized nano drug delivery system was accomplished through *in vitro* experiments, evaluating both its drug release capability and cellular toxicity. Furthermore, the anti-AD activity of HA@Rhy@Hf-MOF, along with its inhibitory effects on Aβ aggregation and tau protein phosphorylation, was assessed through *in vivo* experiments. The schematic diagram of this study is presented in Figure 1.

## 2 Materials and methods

### 2.1 Cell culture

The mouse hippocampal neuron HT22 cell line was purchased from Pricella (Wuhan, China). HT22 cells were cultured in Dulbecco's modified eagle medium (DMEM) supplemented with 10 % fetal calf serum, 100 U/mL penicillin, and 100 μg/mL streptomycin. HT22 cells were incubated at 37°C with a mixture of 95% air and 5% CO_2_.

### 2.2 Animals

Six 8-month-old male wild-type C57BL/6J mice (Control) and 24 8-month-old male C57BL/6J amyloid precursor protein/presenilin 1 (APP/PS1) double-transgenic AD mice were purchased from SiPeiFu (Beijing, China) biotechnology Co., Ltd and were acclimatized for 1 week. Mice were kept in specific pathogen-free animal facilities under constant conditions: room temperature (23 ± 2°C), humidity (60–65%), and a 12 h light-dark cycle.

The mice were divided into Control, AD, Rhy, Hf-MOF, and HA@Rhy@Hf-MOF groups. The Rhy group, Hf-MOF group, and HA@Rhy@Hf-MOF group were intravenously injected with Rhy, Hf-MOF, and HA@Rhy@Hf-MOF, respectively. The dose of Rhy in the free Rhy group and Rhy-loaded Hf-MOF group was equivalent to 10 mg/kg. The Control group and AD group were intravenously injected with the corresponding volume of saline as a control. The administration was carried out once daily for 7 consecutive days. After drug administration, behavioral experiments were conducted. Following the completion of the behavioral experiments, the animals were euthanized, and fresh brain tissues were collected. A portion of the tissues was stored at –80°C for subsequent Western blot experiments, while another portion was fixed in 4% paraformaldehyde for future pathological experiments.

The mice had ad libitum access to standard chow and water.


**Ethical approval:** The research related to animal use has been complied with all the relevant national regulations and institutional policies for the care and use of animals. All animal experiments were approved by Wenzhou Medical University Laboratory Animal Ethics Committee (wydw2023-0641). This study followed the guidelines for the care and use of laboratory animals set forth by the National Research Council of the United States and is reported in accordance with ARRIVE guidelines (https://arriveguidelines.org). The euthanasia method in this study was informed by the American Veterinary Medical Association (AVMA) Guidelines for the Euthanasia of Animals (2020).

### 2.3 Synthesis of Hf-MOF and HA@Rhy@Hf-MOF

Initially, 10 mL of HfCl_4_ solution [2 mg/mL in *N*,*N*-dimethylformamide (DMF)], 10 mL of tetrakis(4-carboxyphenyl)porphyrin solution (5 mg/mL in DMF), and 2 mL of acetic acid were combined in a 40 mL glass vial. The reaction mixture was then stored in an oven at 80°C. Following a 2 h reaction period, 30 mL of dimethyl sulfoxide (DMSO) were added to the reaction system, and after an additional 24 h, the resulting deep purple solid product was obtained through centrifugation. Subsequently, it was washed sequentially with DMSO, a mixture of triethylamine and ethanol (at a volume ratio of 1:20), and ethanol. Finally, freeze-drying was performed to obtain purified Hf-MOF nanoparticles.

For the synthesis of HA@Rhy@Hf-MOF, 413 mg HA were dissolved in 10 mL of deionized water in a 100 mL round-bottom flask, and subsequently, 207 mg 1-ethyl-3-(3-dimethylaminopropyl)carbodiimide hydrochloride were added for activation for 30 m. Then, 200 mg Rhy and Hf-MOF were dissolved in an appropriate amount of DMF, the mixture was introduced into the HA solution, and the reaction was catalyzed by adding a catalytic amount of 4-dimethylaminopyridine. After 4 h of reaction, dialysis was performed using a dialysis bag with a molecular weight cutoff of 3,500: first with DMF for 2 h, then with a mixture of DMF and water (at a volume ratio of 1:1) overnight, and finally with water for 6 h, changing the dialysis water every 2 h. After completion of the dialysis, the reaction solution was transferred to a glass culture dish, pre-frozen in a −20°C refrigerator, and subjected to freeze-drying the next day to obtain HA@Rhy@Hf-MOF.

### 2.4 Characterization of Hf-MOF and HA@Rhy@Hf-MOF

The morphologies and microstructures of Hf-MOF and HA@Rhy@Hf-MOF were characterized by a transmission electron microscope (TEM; FEI Talos F200S, FEI). The crystal structures of Hf-MOF and HA@Rhy@Hf-MOF were analyzed using X-ray diffraction (XRD; Bruker D8 Advance Diffractometer, Berlin). The materials were diluted to 10 μg/mL using deionized water. The particle size distribution and zeta potential of Hf-MOF and HA@Rhy@Hf-MOF were determined using a nanoparticle size analyzer and a zeta potential analyzer (Zetasizer Nano ZS, Malvern), respectively.

### 2.5 Drug release study of HA@Rhy@Hf-MOF

The prepared HA@Rhy@Hf-MOF was diluted to a concentration of 1 mg/mL using phosphate buffered saline (PBS). After 0, 2, 6, 10, 20, and 30 h of shaking and stirring, the isolate was filtered through a filter membrane (0.45 μm) (FF397, Beyotime) to obtain the separated solutions at different time points. The released Rhy was analyzed using High Performance Liquid Chromatography (HPLC; Agilent 1260, Agilent), and the release curves were plotted.

### 2.6 Cell viability assay

The cell viability was assessed using Cell Counting Kit-8 (CCK-8) assay (Beyotime, China). The HT22 cells (1 × 10^4^ cells) were seeded in 96-well plates and treated with Rhy, Hf-MOF, and HA@Rhy@Hf-MOF of various concentrations. Subsequently, the cells underwent PBS buffer washes and were then exposed to a CCK-8 solution combined with DMEM for 1 h at 37°C. The absorbance at 450 nm was assessed to ascertain cell viability. Cell viability = [OD (treated) − OD (blank)]/[OD (control) − OD (blank)] × 100%.

### 2.7 Morris water maze (MWM) test

The impact of HA@Rhy@Hf-MOF on spatial learning and memory were evaluated in APP/PS1 mice through the MWM test. The testing arena was configured to be 120 cm in diameter and housed escape platforms measuring 10 cm in diameter, imperceptible from the water’s surface. Water temperature was meticulously maintained at 22 ± 1°C, supplemented with non-toxic titanium dioxide. The experimental protocol encompassed a 1-day platform visibility phase, followed by a 4-day training regimen, and concluded with a 1-day probe trial. Each mouse was allotted 1 min to locate the platform during each training session, which was repeated four times daily. During the examination phase, the platform was removed, and the mice were introduced into the water maze for unrestricted swimming for a duration of 90 s. Subsequently, the mice underwent a probe trial to evaluate the number of platform crosses, and the duration of their stay as well as the movement distance in the target quadrant. The movements of the mice in the maze were analyzed using the ANYmaze video-tracking system. There were six mice in each group, and each mouse was tested three times.

### 2.8 Novel object recognition (NOR) test

NOR test served as a method for assessing learning and memory, leveraging the innate tendency of mice to explore novel objects. The experimental setup comprised a rectangular box containing three objects labeled “a,” “b,” and “c.” Objects “a” and “b” were identical, while object “c” distinctly differed from both “a” and “b.” Over the course of the first and second days, each mouse (*n* = 6) undergoing assessment was acclimated to the environment for 10 min. On the third day, we introduced the mouse into an opaque box with two objects labeled “a” and “b” placed beside it, allowing them to explore for 5 min (training phase, T1). Subsequently, the mouse was returned to its cage. After a 1-h interval, object “b” was replaced with object “c” in the same location, and the mouse was reintroduced to the box for another 5 min of exploration (testing phase, T2). During the experiment, these areas and objects were cleaned with ethanol to ensure proper hygiene. The time spent exploring familiar (*F*) and novel (*N*) objects in T2 was recorded, respectively. Discrimination index = (*N* − *F*)/(*N* + *F*) × 100%. There were six mice in each group, and each mouse was tested three times.

### 2.9 Elevated plus maze (EPM) test

The standard EPM test was employed to assess the impact of HA@Rhy@Hf-MOF on anxiety-like behavior of mice, as previously described [18]. The duration spent in the closed and open arms were quantified utilizing the ANY-maze software. Increased activity in the open arms was construed as indicative of anxiolitic behavior. The shorter the time the mice spent in the open arms, the more severe their anxiety. This study recorded the time mice spent in the open arms and the total time, reflecting the experimental results using a ratio (percentage of time spent by mice in the open arms compared to the total time). There were six mice in each group, and each mouse is tested three times.

### 2.10 Hematoxylin-eosin (HE) staining

The brains of mice were harvested and immersed in 4% formaldehyde solution, where they underwent fixation overnight at 4°C. Following fixation, the tissues were dehydrated and embedded in paraffin before being sectioned into slices of 5 μm thickness. These sections were then deparaffinized using xylene, followed by rehydration through an ethanol gradient. Subsequently, the tissue sections were stained using a HE Staining Kit (Solarbio, Beijing, China) in the appropriate sequence. The histopathological alterations within the hippocampus were observed using an optical microscope (Nikon eclipse Ni, Japan) at 400× magnification.

### 2.11 Nissl staining

The brain sections were subjected to dewaxing and rehydration procedures, followed by microwave treatment in 0.01 M sodium citrate buffer for 5 min. Subsequently, the sections were allowed to cool to room temperature and rinsed thrice with PBS. After staining with toluidine blue, the sections underwent dehydration in 95% ethanol for 5 min, followed by immersion in 100% ethanol for 10 min. Then, the sections were rendered transparent using xylene. After drying, the sections were sealed in neutral resin. The hippocampal neuronal apoptosis was visualized with an optical microscope at 400× magnification.

### 2.12 Immunofluorescence (IF)

The hippocampus tissues were initially fixed in 4% paraformaldehyde, followed by dehydration in a gradient of ethanol and permeabilization using xylene. Subsequently, the tissues were embedded in paraffin and sliced into sections with a thickness of 5 μm. These sections underwent deparaffinization in xylene and rehydration in a gradient of ethanol.

After dewaxing and hydration, the slices were immersed in citrate buffer and subjected to high-pressure repair. Following the high-pressure repair, the slices were allowed to return to room temperature and blocked with goat serum sealing solution for 1 h. Subsequently, the slices were incubated with a specific primary antibody Anti-Beta Amyloid Antibody (PB9091, Boster) [19] pre-diluted at a ratio of 1:200, at 4°C overnight.

Following thorough washing, the sections were incubated for 1 h at room temperature with Anti-rabbit IgG (H + L), F(ab’)2 Fragment (Alexa Fluor 488 Conjugate)^®^ (1∶1,000) (#4412, Cell Signaling Technology) [20]. After rinsing in PBS, the slices were stained with 4′,6-diamidino-2-phenylindole and examined under a fluorescence microscope.

### 2.13 Western blotting analysis

Separate the hippocampal tissue from mice and extract total proteins from the hippocampal tissue using radioactive immunoprecipitation assay buffer. Equal amounts of total protein were separated via 10% sodium dodecyl sulfate-polyacrylamide gel electrophoresis, after which they were transferred onto polyvinylidene fluoride membranes for blotting. The membrane was then blocked with 5% skimmed milk in Tris-buffered saline at room temperature for 1 h. Subsequently, it was incubated with primary antibodies Beta Amyloid Polyclonal antibody (1:1000) (25524-1-AP, Proteintech)[21], Anti-Tau (phospho S396) antibody (1:1000) (ab32057, abcam) [22], Phospho-TAU (Ser202/Thr205) Recombinant antibody (82568-1-RR, Proteintech) (1:5,000) [23] Anti-Tau (phospho T231) (1:2,000) (ab151559, abcam) [24], TAU Monoclonal antibody (66499-1-Ig, Proteintech) (1:3,000) [25], and GAPDH Polyclonal antibody (1:5,000) (10494-1-AP, Proteintech) [26] at 4°C overnight. Following this, it was incubated with Horseradish peroxidase-conjugated goat anti-rabbit IgG (H + L) (A0208, Beyotime) [27] or Horseradish peroxidase-conjugated goat anti-mouse IgG (H + L) (A0216, Beyotime) [28] at room temperature for 2 h. Detection of protein bands was facilitated using a chemiluminescence substrate, and the Image J software was employed for semi-quantitative analysis.

### 2.14 Statistical analysis

Collect experimental data independently repeated at least three times and import the data into GraphPad Prism 8 software for statistical analysis. The measured data were presented as mean value ± standard deviation. One-way analysis of variance (ANOVA) was used for comparisons among multiple groups. A *p*-value less than 0.05 was considered statistically significant.

## 3 Results

### 3.1 Synthesis and characterization of HA@Rhy@Hf-MOF

**Figure 2:**
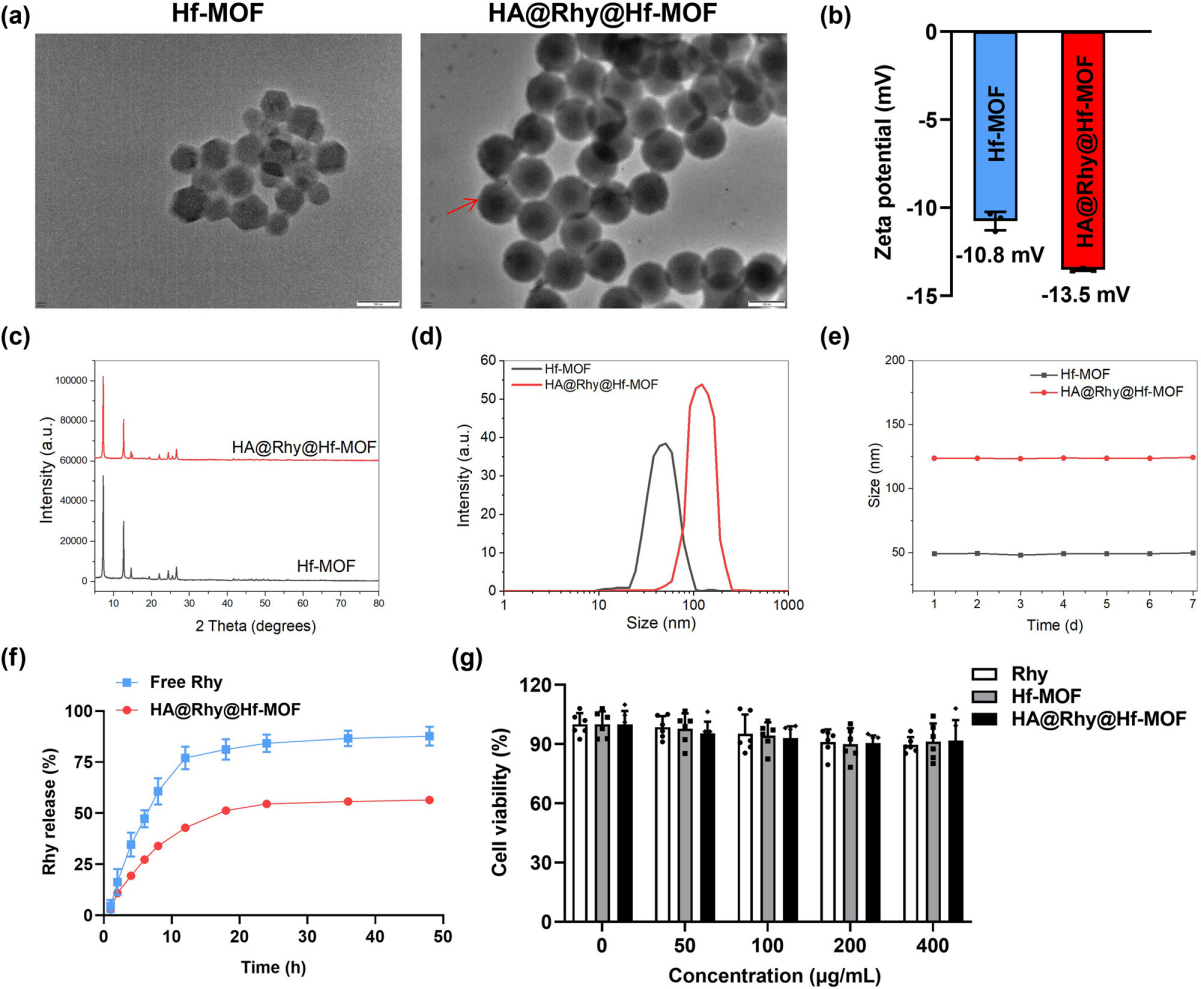
Characterization of HA@Rhy@Hf-MOF. (a) The morphology of Hf-MOF and HA@Rhy@Hf-MOF determined by TEM. (b) The zeta potential of Hf-MOF and HA@Rhy@Hf-MOF measured via zeta potential analyzer. (c) The average particle size distribution. (d) seven-day average particle size of Hf-MOF and HA@Rhy@Hf-MOF evaluated with nanoparticle size analyzer. (e) The crystal structures of Hf-MOF and HA@Rhy@Hf-MOF detected by XRD. (f) Drug release rate of Rhy with/without nanomaterial encapsulation. (g) Cell viabilities were assessed using CCK-8 assay following 24-h incubation with varying concentrations of Rhy, Hf-MOF, and HA@Rhy@Hf-MOF, respectively. The ANOVA results show: (Rhy), *F* (4, 25) = 2.896; (Hf-MOF), *F* (4, 25) = 1.822; (HA@Rhy@Hf-MOF), *F* (4, 25) = 1.752. *N* = 6.

As shown in Figure 2a, Hf-MOF exhibited regular hexagonal particles, while after HA modification, it displayed a uniform and round shape with a transparent coating of HA visible on the surface. Zeta potential analysis revealed that both Hf-MOF and HA@Rhy@Hf-MOF carried negative charges (Figure 2b). XRD results indicated that the loading of Rhy and the HA modification did not affect the crystal structures of Hf-MOF (Figure 2c). Moreover, the mean particle size of Hf-MOF was approximately 49.2 nm, as measured by Mastersizer, and the nanoparticle size increased to about 123.7 nm after HA modification (Figure 2d). After seven consecutive days of measurements under 4°C, the particle sizes of both Hf-MOF and HA@Rhy@Hf-MOF showed no significant changes, indicating the excellent stability of these nanoparticles (Figure 2e).

### 3.2 Drug release and cytotoxicity of HA@Rhy@Hf-MOF

*In vitro* drug release results indicated that the free Rhy reached a release rate of 60% within 10 h, exhibiting a burst release phenomenon, which is unfavorable for sustained drug circulation. In contrast, the drug release from the HA@Rhy@Hf-MOF nanoparticles were approximately 40% within the first 10 h and reached around 50% after 20 h, suggesting a sustained and gradual drug release behavior (Figure 2f). The cytotoxicity of different concentrations of Rhy, Hf-MOF, and HA@Rhy@Hf-MOF was evaluated in HT22 cells following a 24-h incubation period using the CCK-8 assay (Figure 2g). Our findings indicated that all the particles had no discernible impact on cell viabilities up to a concentration of 400 μg/mL, which represents a relatively high concentration. From this, it can be inferred that free Rhy itself possesses low toxicity, and both Hf-MOF and HA@Rhy@Hf-MOF exhibit biocompatibility and maintain relatively low toxicity profiles.

### 3.3 HA@Rhy@Hf-MOF ameliorates cognitive deficiency, spatial learning, and anxiety in APP/PS1 mice

**Figure 3:**
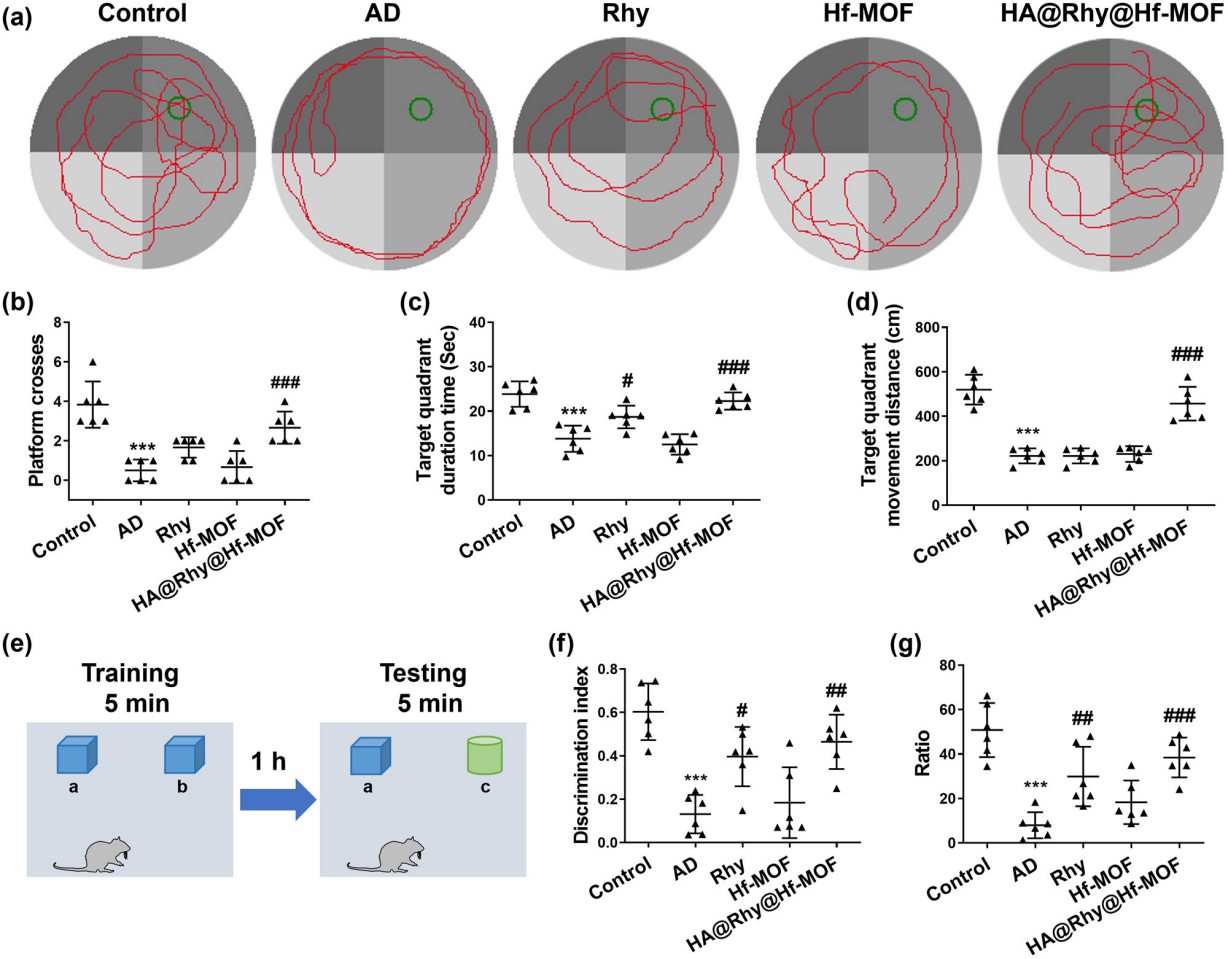
Behavioral experimental indicators in mice. (a)–(d) The MWM test is used to assess the learning and memory capabilities of mice. (e) and (f) The NOR test is used to evaluate the cognitive memory ability of mice. (g) The EPM test is used to assess the impact of anxiety on mice. The ANOVA results show: (b) *F* (4, 25) = 18.04; (c) *F* (4, 25) = 23.31; (d) *F* (4, 25) = 46.68; (f) *F* (4, 25) = 13.48, and *G* (4, 25) = 15.62. *N* = 6. ***P < 0.001 vs Control; ^#^ *P* < 0.05, ^##^ *P* < 0.01, ^###^ *P* < 0.001 vs AD.

The MWM test and NOR test were conducted to assess the impact of HA@Rhy@Hf-MOF on spatial learning and memory capabilities in APP/PS1 transgenic mice. The representative trajectories of mice in each group during the MWM test are depicted in Figure 3a. Notably, mice in the AD group exhibited a significant decrease in the number of platform crossings (*P* < 0.001, Figure 3b), target quadrant duration time (*P* < 0.001, Figure 3c), and target quadrant movement distance (*P* < 0.001, Figure 3d), in comparison to the control group. Nevertheless, AD mice treated with free Rhy displayed a marked increase in the number of platform crossings (*P* < 0.01) and the target quadrant duration time (*P* < 0.05), without inducing a significant alteration in the target quadrant movement distance. Moreover, the improvement in cognitive and behavioral abilities of the AD mice was further enhanced in the HA@Rhy@Hf-MOF group, with the target quadrant movement distance for mice in the HA@Rhy@Hf-MOF group being significantly higher compared to the AD group (*P* < 0.001, Figure 3d). The procedure employed for the NOR test is delineated in Figure 3e. In the NOR test, the discrimination index served as an indicator for assessing learning and memory ability in mice, with a higher index value signifying a greater preference for the new object. Mice in the control group exhibited a preference for the novel objects, whereas the mice in the AD group displayed no significant preference. The administration of Rhy and HA@Rhy@Hf-MOF notably restored the impaired learning and memory capabilities of APP/PS1 transgenic mice, as evidenced by discrimination values that significantly exceeded those of the AD group (*P* < 0.01, *P* < 0.001, Figure 3f), where the efficacy of HA@Rhy@Hf-MOF was superior to free Rhy. Furthermore, EPM test depicted the percentage of time spent in the open arms relative to the total duration for each group of mice (Figure 3g). AD mice exhibited a significant reduction in activity in the open arms (*P* < 0.001), confirming an increased anxiety-like behavior. Treatment with Rhy alleviated the mice’s anxiety levels, indicating a positive effect of Rhy in reducing anxiety-related behavior. Notably, the reversal effect was more pronounced with HA@Rhy@Hf-MOF.

### 3.4 HA@Rhy@Hf-MOF decreases hippocampus neurons damage in APP/PS1 mice

**Figure 4:**
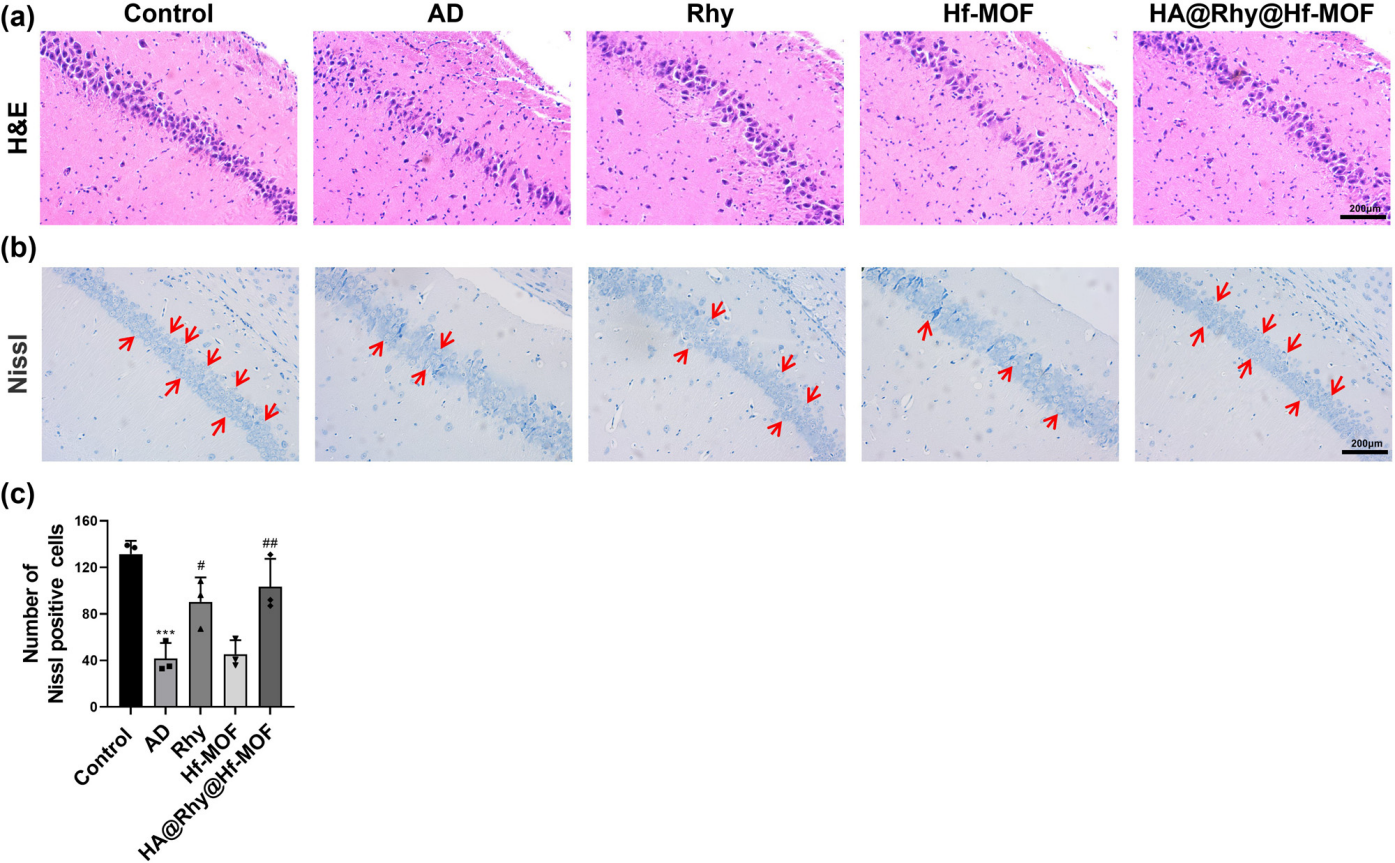
Pathomorphological examination of the mice hippocampus. (a) HE staining and (b) and (c) Nissl staining are used to observe neuropathological changes in neural tissue; *N* = 3.

Following the identification of behavioral changes, a pathomorphological examination of the mouse hippocampus was conducted. Results from HE staining revealed that in the control group, neurons in the CA1 region of the hippocampus exhibited orderly arrangement and normal structure. In contrast, the AD group showed significant pathological changes, with disorganized neuronal arrangement and loose structure. Conversely, the AD group exhibited a substantial number of swollen neurons with a loose structure, karyopyknosis, and the formation of vacuolar structures. In comparison to the AD group, the pathological alterations in hippocampal neurons were markedly ameliorated in the HA@Rhy@Hf-MOF group, while free Rhy treatment only exhibited slight improvement (Figure 4a). Subsequently, the roles of Rhy in hippocampal neuron survival were investigated through Nissl staining (Figure 4b and c). Hippocampal neurons in the control group displayed a full shape, regular arrangement, and normal density, while the number of positive neurons cells in the CA1 region of the hippocampus significantly decreased in AD mice. Following treatment with HA@Rhy@Hf-MOF, the number of positive neurons cells in hippocampus was significantly increased compared to the AD group, whereas free Rhy displayed a limited restorative effect. All these results collectively suggest that HA@Rhy@Hf-MOF can mitigate damage to hippocampal neurons in AD mice.

### 3.5 HA@Rhy@Hf-MOF reduced hippocampal Aβ deposition and tau phosphorylation in APP/PS1 mice

**Figure 5:**
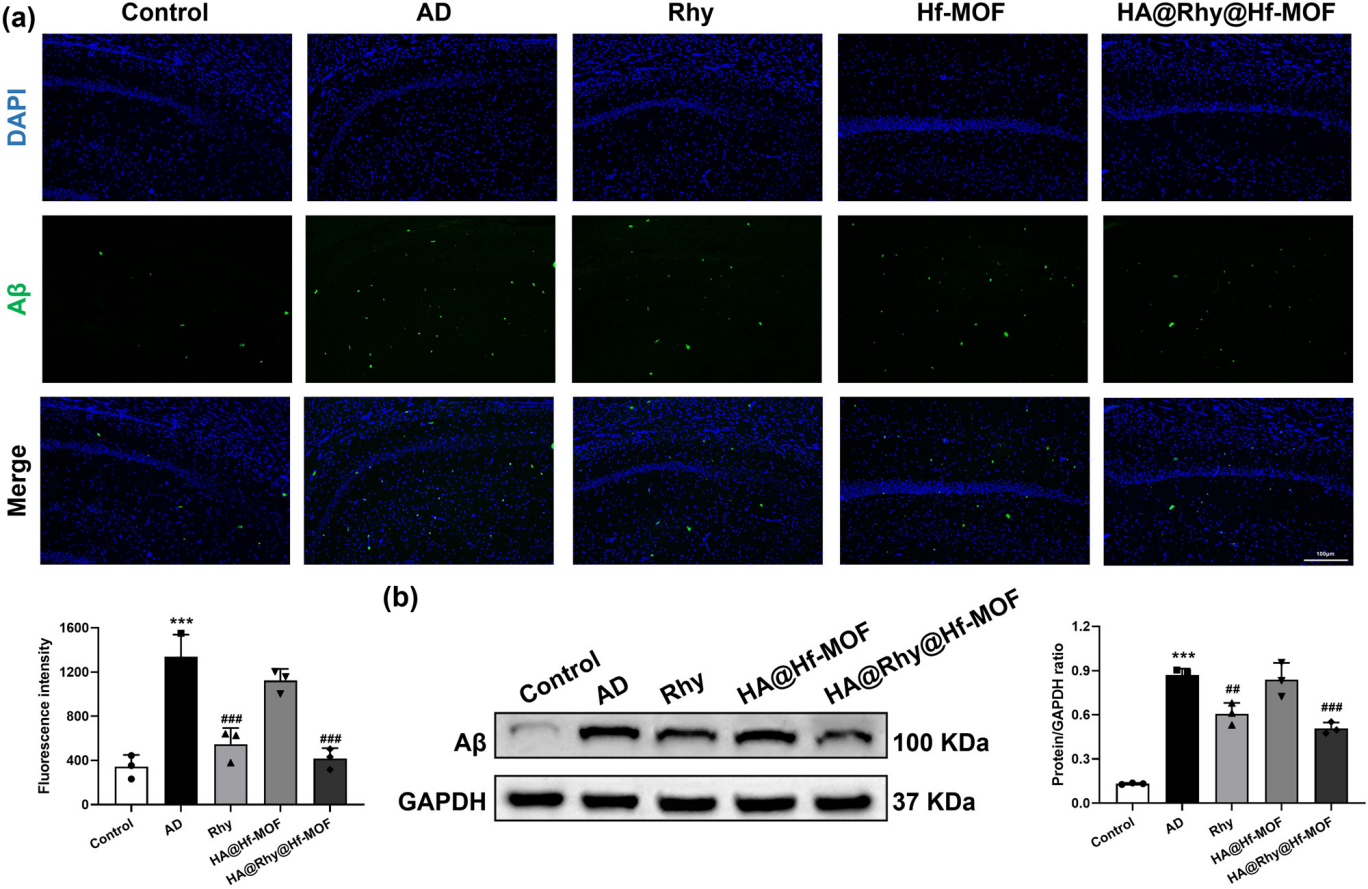
Aβ plaques deposition in mice hippocampus. (a) IF staining and (b) Western blot are used to detect the expression levels of Aβ in mouse hippocampal tissue. The ANOVA results show: (a) *F* (4, 25) = 32.28; (b) *F* (4, 25) = 59.83. *N* = 3. ***P < 0.001 vs Control; ^##^ *P* < 0.01, ^###^ *P* < 0.001 vs AD.

**Figure 6:**
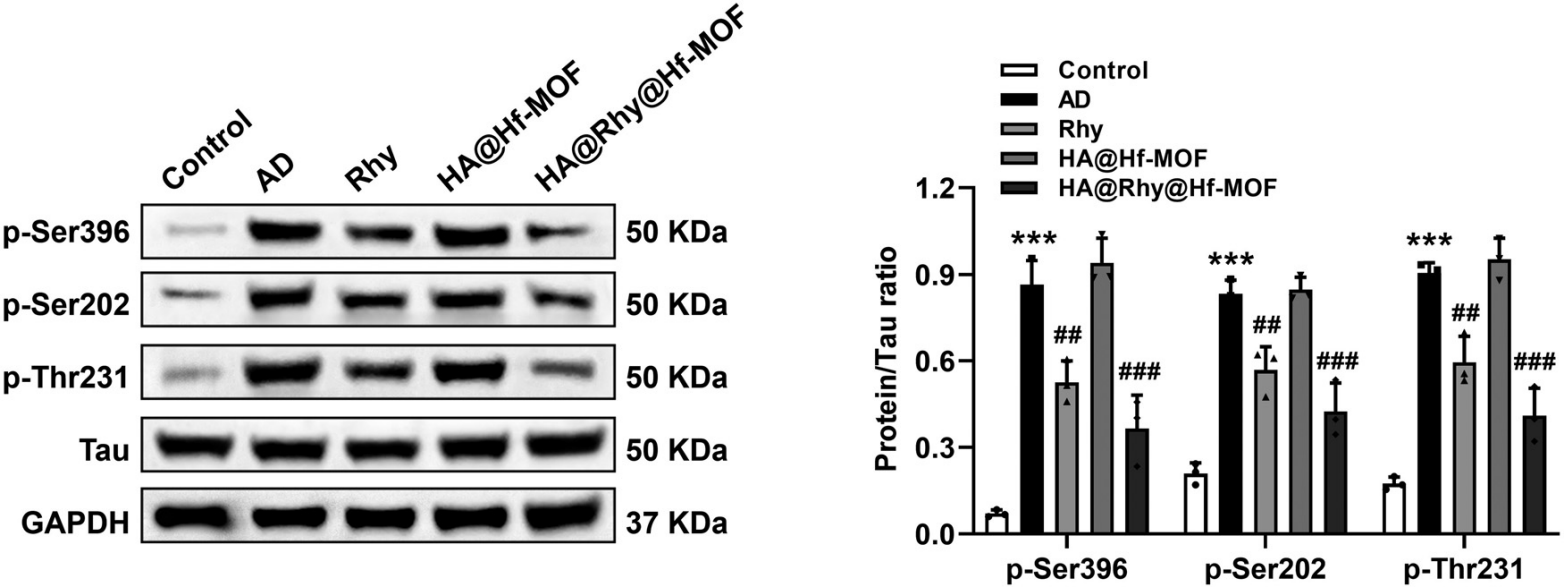
The hippocampal protein levels of total Tau and phosphorylated Tau at several sites, including Ser396, Ser202, and Thr231 determined by Western blot. The ANOVA results show that for (p-Ser396): *F* (4, 25) = 57.78; (p-Ser202): *F* (4, 25) = 51.89; (p-Thr231): *F* (4, 25) = 66.75. ***P < 0.001 vs Control; ^##^ *P* < 0.01, ^###^ *P* < 0.001 vs AD. *N* = 3.

To explore the potential of HA@Rhy@Hf-MOF in mitigating the formation of Aβ plaques associated with AD in mice, IF staining and Western blot analyses were conducted. Clearly, in hippocampus of APP/PS1 mice, the plaques immune-stained with Aβ exhibited a notable increase compared to the control group. Notably, the Rhy group displayed a moderate reduction in these effects, while the HA@Rhy@Hf-MOF group showcased the most conspicuous reduction in this pathological manifestation (Figure 5a). Western blot experiments further confirmed above results, revealing that the markedly elevated Aβ protein levels in AD mice were successfully and significantly mitigated by HA@Rhy@Hf-MOF (Figure 5b). The hyperphosphorylation of Tau protein is implicated in neuronal dysfunction during the progression of AD [29]. As reported, major biomarkers for AD include the senile plaques of Aβ peptide precipitates and the neurofibrillary tangles (NFTs) of fibrillar hyperphosphorylated tau protein [30,31]. NFTs are composed of fibrils of abnormally phosphorylated tau protein. Among them, phosphorylated Tau at several sites, including Ser396, Ser202, and Thr231, have been found to be associated with the pathological progression of AD [30,31]. Evaluating the hippocampal protein levels of total Tau and phosphorylated Tau at various sites, such as Ser396, Ser202, and Thr231, can reflect the condition of AD. Analysis through Western blotting unveiled a noteworthy elevation in Tau protein phosphorylation at all three aforementioned sites in the AD-induced condition (*P* < 0.001, Figure 6). Importantly, both Rhy and HA@Rhy@Hf-MOF exhibited inhibitory effects on the abnormal phosphorylation of Tau (*P* < 0.01, *P* < 0.001), with the efficacy of HA@Rhy@Hf-MOF surpassing that of free Rhy.

## 4 Discussion

AD stands out as the most prevalent form of dementia, marked by a cognitive decline that notably impacts memory and judgment. With a progressively aging population, AD has emerged as a significant public health concern [32]. The prevailing theory posits that an imbalance between the production and clearance of Aβ serves as the initiating factor, playing a crucial role in triggering other observed abnormalities in AD, while the tau protein emerges as a subsequent pathological event, further exacerbating neurodegeneration thereafter [33]. In the current study, a substantial accumulation of Aβ and phosphorylation of tau were found in the brains of APP/PS1 mice. Here the HA-modified MOF loading with Rhy was engineered, aiming to assess and contrast the therapeutic effect of free Rhy and HA@Rhy@Hf-MOF on AD mice.

Accumulating evidence underscores the beneficial impact of naturally occurring alkaloids derived from plants in mitigating neurodegenerative disorders [34]. Recent research has elucidated the protective effects of Rhy across various models of neurotoxicity [35]. Crucially, Rhy has demonstrated a significant capacity to mitigate cellular death and hyperphosphorylation of tau protein in the AD cellular model [35]. Consistent with findings from prior studies, our present investigation affirms that free Rhy could partially ameliorate memory loss and neuronal damage, coupled with a reduction in Aβ aggregation and tau phosphorylation in APP/PS1 mice. Nonetheless, the anticipated potent effect of free Rhy was not realized, primarily due to its inherent limitations stemming from low water solubility and poor bioavailability upon peripheral administration. Hf-MOF generates substantial amounts of singlet oxygen, which is instrumental in inhibiting Aβ aggregation. Furthermore, through further HA modification, the water solubility of the nano drug delivery system was enhanced, presenting added benefits compared to the administration of free Rhy for the treatment of AD. Characterization results from this experiment indicated that HA@Rhy@Hf-MOF boasts nanoscale diameter, structural stability, sustained drug release, low toxicity, and high biocompatibility, suggesting that it is a highly suitable nano drug delivery system for Rhy.

To further assess the *in vivo* effects of HA@Rhy@Hf-MOF, 8-month-old APP/PS1 transgenic mice were utilized, modeling the pathological progression of AD in humans [36]. The MWM test was employed to investigate spatial learning and memory in these animals following hippocampal damage. This assessment stands as a valuable instrument for assessing cognitive impairment in animal models of AD [37]. During the probe trial, the AD group displayed a significant decrease in platform crosses, duration time in the target quadrant, and movement distance in the target quadrant compared to the control group. These findings indicated a noticeable decline in spatial learning and memory in the APP/PS1 mice, closely resembling the cognitive impairment observed in AD [38]. Importantly, the administration of HA@Rhy@Hf-MOF substantially mitigated these observed changes, highlighting its effectiveness in enhancing the cognitive abilities of APP/PS1 mice. Moreover, the NOR test and EPM test further confirmed the beneficial effects of HA@Rhy@Hf-MOF on spatial learning and anxiety in AD mice.

It has been documented that the CA1 region in the hippocampus exhibits particular vulnerability to the neurotoxic effects induced by Aβ, potentially inducing degenerative lesions in the hippocampus [39]. Consistent with this notion, our ongoing anatomical analyses employing HE staining and Nissl staining reveal new evidence of abnormalities within the hippocampal region, particularly focusing on the CA1 region, in APP/PS1 mice. The staining results demonstrated a significantly improved pathological condition and a higher neuron count in the HA@Rhy@Hf-MOF group compared to the AD group and free Rhy group, indicating the protective efficacy of HA@Rhy@Hf-MOF on neurons. These findings are in accordance with our previous study and further reinforce the beneficial effects of HA@Rhy@Hf-MOF in the treatment of AD. Furthermore, among the numerous complex and unclear mechanisms underlying AD, the most widely recognized is the amyloid cascade hypothesis, which states that the abnormal aggregation and deposition of Aβ in the brain, along with the hyperphosphorylation of tau protein, serve as the initiating factors and key events in AD, triggering a series of abnormal lesions [40]. Upon Aβ stimulation, there is a noticeable surge in the hyperphosphorylation of tau protein at the AD-associated epitope and paired helical filament, leading to cytoskeletal destabilization, memory impairment, and neuronal demise [35]. Consequently, inhibiting the deposition of Aβ plaques and the phosphorylation of tau are deemed effective strategies for the treatment of AD. Previous studies have shown that phosphorylated Tau at Ser396, Ser202, and Thr231 is closely associated with the severity of AD neuronal cell pathology [30,31]. In our study, AD mice displayed pronounced plaques immunostained with Aβ, heightened expression of Aβ protein, and elevated site-specific phosphorylation of Tau (Ser396, Ser202, and Thr231), aligning with the typical pathology of AD. Additionally, Rhy alleviated the aforementioned adverse situations to a certain extent, while under the condition of the HA@Rhy@Hf-MOF nano drug delivery system, it exhibited a more potent effect. These outcomes implied that HA@Rhy@Hf-MOF exhibits effectiveness in treating AD by targeting the deposition of Aβ plaques and inhibiting site-specific phosphorylation of Tau, thereby exerting protective effects on neurons.

## 5 Conclusion

Overall, Rhy is indeed a safe drug that can effectively improve AD. With the assistance of the nano-system, HA@Rhy@Hf-MOF can increase its bioavailability, thereby enhancing its therapeutic efficacy. Treatment with HA@Rhy@Hf-MOF yields improvements in cognitive deficiency, spatial learning, and anxiety. Moreover, it mitigates damage to hippocampal neurons while concurrently reducing Aβ deposition and tau phosphorylation in the hippocampus of APP/PS1 mice. These findings not only present a promising avenue for disease-modifying treatments in AD but also generate heightened anticipation for the potential application of this nano-drug delivery system in addressing other intricate neurodegenerative diseases.
